# Staged subthalamotomy

**DOI:** 10.1186/2050-5736-3-S1-O6

**Published:** 2015-06-30

**Authors:** Jeff Elias

**Affiliations:** 1 University of Virginia, Charlottesville, VA, United States

## Background/introduction

A clinical trial is being planned to investigate the management of medication-refractory motor symptoms associated with Parkinson’s disease using a unilateral, focused ultrasound subthalamotomy performed in a staged fashion.

## Methods

For this study, ten subjects with medication-refractory symptoms or side effects of advanced Parkinson’s disease will be enrolled. Each patient will be treated with a “sub-therapeutic” (stage 1) focused ultrasound subthalamotomy and observed for thirty days.

Those who develop severe and involuntary movements, such as hemiballismus, will be excluded from the second stage procedure. For those who tolerate subthreshold lesioning, a second, full subthalamotomy ablation (stage 2) with focused ultrasound will be performed. Validated Parkinson’s disease rating scales, cognitive assessments, and MRI will be obtained before and after the procedures.

